# Identical bacterial populations colonize premature infant gut, skin, and oral microbiomes and exhibit different in situ growth rates

**DOI:** 10.1101/gr.213256.116

**Published:** 2017-04

**Authors:** Matthew R. Olm, Christopher T. Brown, Brandon Brooks, Brian Firek, Robyn Baker, David Burstein, Karina Soenjoyo, Brian C. Thomas, Michael Morowitz, Jillian F. Banfield

**Affiliations:** 1 Department of Plant and Microbial Biology, University of California, Berkeley, California 94720, USA;; 2 Department of Surgery, University of Pittsburgh School of Medicine, Pittsburgh, Pennsylvania 15213, USA;; 3 Division of Newborn Medicine, Children's Hospital of Pittsburgh and Magee-Womens Hospital of UPMC, Pittsburgh, Pennsylvania 15213, USA;; 4 Department of Earth and Planetary Science, University of California, Berkeley, California 94709, USA;; 5 Department of Environmental Science, Policy, and Management, University of California, Berkeley, California 94720, USA;; 6 Earth Sciences Division, Lawrence Berkeley National Laboratory, Berkeley, California 94720, USA

## Abstract

The initial microbiome impacts the health and future development of premature infants. Methodological limitations have led to gaps in our understanding of the habitat range and subpopulation complexity of founding strains, as well as how different body sites support microbial growth. Here, we used metagenomics to reconstruct genomes of strains that colonized the skin, mouth, and gut of two hospitalized premature infants during the first month of life. Seven bacterial populations, considered to be identical given whole-genome average nucleotide identity of >99.9%, colonized multiple body sites, yet none were shared between infants. Gut-associated *Citrobacter koseri* genomes harbored 47 polymorphic sites that we used to define 10 subpopulations, one of which appeared in the gut after 1 wk but did not spread to other body sites. Differential genome coverage was used to measure bacterial population replication rates in situ. In all cases where the same bacterial population was detected in multiple body sites, replication rates were faster in mouth and skin compared to the gut. The ability of identical strains to colonize multiple body sites underscores the habit flexibility of initial colonists, whereas differences in microbial replication rates between body sites suggest differences in host control and/or resource availability. Population genomic analyses revealed microdiversity within bacterial populations, implying initial inoculation by multiple individual cells with distinct genotypes. Overall, however, the overlap of strains across body sites implies that the premature infant microbiome can exhibit very low microbial diversity.

Infants are born near sterile and continually acquire microbial colonists until reaching an adult-like state at around 2–3 yr of age (Cilieborg et al. 2012; Faith et al. 2015). The microbiota during the first 100 d of life is especially important, as dysbiosis during this “critical window” has been linked to a number of problems later in life, especially relating to the developing immune system (Costello et al. 2012; Cahenzli et al. 2013; Sim et al. 2013; Arrieta et al. 2015). The nature of dysbiosis during the critical window has yet to be clearly defined, but a number of studies have implicated low-diversity as a marker (Cahenzli et al. 2013; Arrieta et al. 2015). Initial colonists are acquired maternally and from the immediate environment, but early life clinical factors (such as birth by cesarean section and neonatal antibiotic administration) can disrupt the normal acquisition process (Ding and Schloss 2014; Bäckhed et al. 2015; Mueller et al. 2015). Among premature infants, who generally harbor microbial communities of limited diversity and instability (Costello et al. 2013; Sim et al. 2013; Ward et al. 2016), this disruption can lead to colonization by resident microbes of the neonatal intensive care unit (NICU) (Brooks et al. 2014; Shin et al. 2015).

Operational taxonomic units (OTUs) identified from 16S rRNA hypervariable region surveys have approximately genus-level resolution (Tu et al. 2014; Jovel et al. 2016). Using this methodology, it has been suggested that, within 24 h after birth, the microbiomes of the mouth, skin, and gut are undifferentiated (Dominguez-Bello et al. 2010) and that site-specific communities develop over the first weeks of life (Costello et al. 2013; Dominguez-Bello et al. 2016). This could imply a common inoculum to all body sites followed by body site-specific selection and immigration. However, even organisms with identical 16S rRNA sequences have been shown to have different genomic and functional profiles (Prosser et al. 2007; Achtman and Wagner 2008; Luo et al. 2015). Such differences could imply different inoculum sources and processes as well as differences in antibiotic susceptibility and strain complexity. Further, if bacterial populations occupy multiple sites, a strain eliminated from one body site could be replaced by dispersal of the same strain from another site (Costello et al. 2012). This could contribute to both pathogen persistence and retention of founding “keystone species” (Dominguez-Bello et al. 2016). Clearly, more sensitive methods like whole-genome sequencing (considered the gold standard of strain typing) (Snitkin et al. 2012) are needed to determine if strains are the “same” or “different.”

Genome-resolved methods have yet to be widely applied to the human microbiome, and thus the level of microdiversity present within human-associated microbial populations is largely unknown. Strain-level diversity is common across other ecosystems and is hypothesized to contribute to the stability of populations of related organisms in the face of phage predation and changing environmental conditions (Jaspers and Overmann 2004; Erkus et al. 2013; Sharon et al. 2013). Methods based on assembly-free metagenomics have attempted to document strain diversity, but reliance on reference genome sequences limits identification of strains to those that have already been analyzed. Other methods have been proposed to document deviations from reference strain sequences, including ConStrains (Luo et al. 2015) and PanPhlan (Ward et al. 2016), but these methods only consider portions of the genome (specific marker genes and coding regions, respectively) and thus fall short of the resolving power needed to account for small-scale differences (Snitkin et al. 2012). While requiring more computational time and manual curation, genome-resolved metagenomics has been used to successfully investigate strain-level differences in the infant gut microbiota several times (Morowitz et al. 2011; Sharon et al. 2013; Raveh-Sadka et al. 2015) and, in combination with previously developed methods (Lang et al. 2013; Luo et al. 2015), has the potential to identify subpopulations of microbes that differ by even a single nucleotide.

A recent study by Browne et al. (2016) found that over half of microbes in the human gut can enter nonvegetative states. This is an important fact to consider when interpreting the studies referenced above, as while the same microbes may be present in different environments, their activity levels in distinct body sites have yet to be investigated. A number of laboratory methods have been developed to discriminate between live and dead cells, including the use of propidium monoazide (Nocker et al. 2006), redox sensing probes (Rodriguez et al. 1992), and the incorporation of radioactive substrates (Karl 1979). However, these methods have limited ability to discriminate between levels of activity, require extensive testing for use with different organisms, and would be difficult to perform in the context of human hosts. An attractive solution is the utilization of differential genome coverage, a method recently described by Korem et al. (2015), that measures the fraction of the bacterial population currently undergoing active DNA replication. However, assembly errors in reference genome databases (Salzberg and Yorke 2005) and divergence among microbial genomes (Tenaillon et al. 2010; Luo et al. 2011; Rosen et al. 2015; King et al. 2016) cast doubt on methods that map directly to genomes in reference collections. Using draft genomes recovered from the samples themselves would solve this problem, but as circular genomes are only rarely recovered, a method to determine the order of the contigs before calculation of growth rates is needed.

## Results

### Community profile

**Figure 1.**
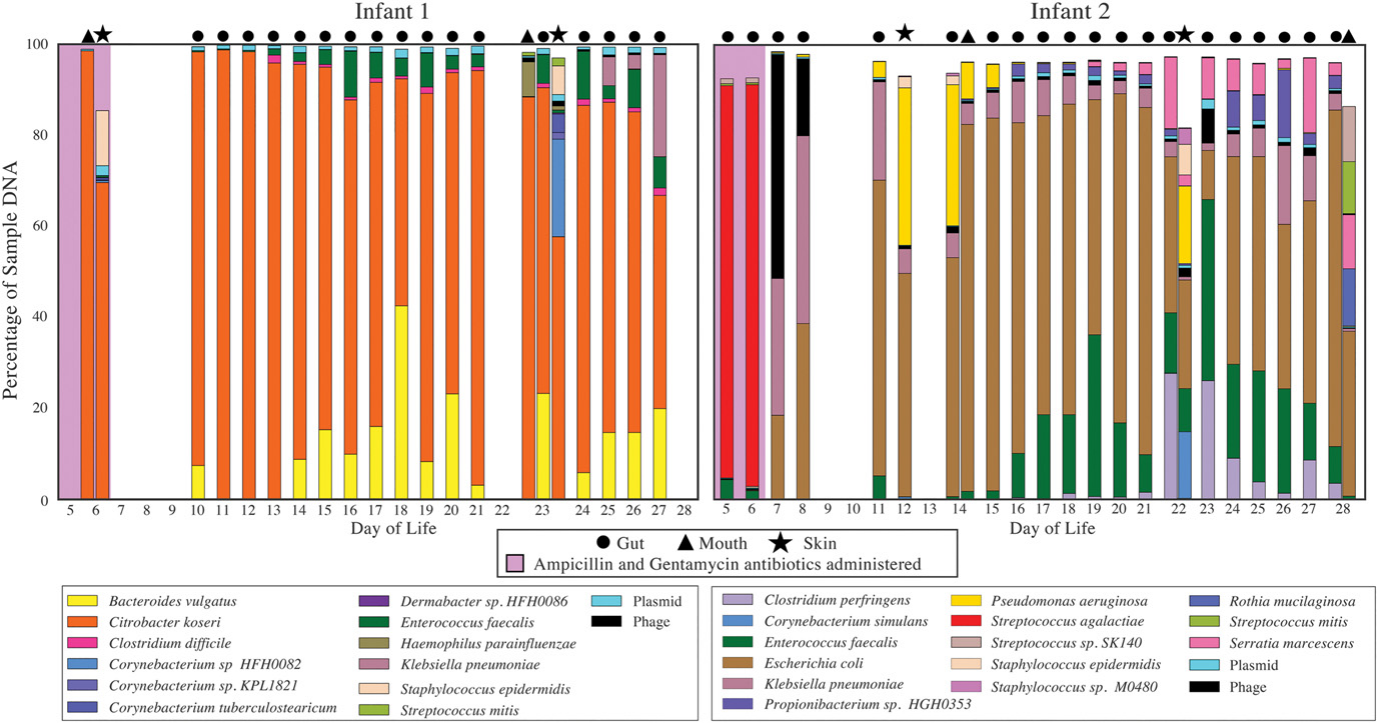
Compositional profile of microbial communities colonizing the mouth, skin, and gut of two premature infants. Each colored box represents the percentage of nonhuman reads mapping to an assembled genome, and the stacked boxes for each sample show the fraction of the reads in that data set accounted for by the genomes from that sample.

Two premature infants were recruited for this study with parental consent at Magee-Womens Hospital of the University of Pittsburgh Medical Center. Clinical information for these infants is summarized in Supplemental Table S1. Both infants were born via vaginal delivery to women with pregnancies complicated by chorioamnionitis. Each infant was treated immediately after birth with 7 d of initial antibiotic treatment (ampicillin and gentamycin). We collected 17 and 20 fecal samples during the first month of life for the two infants (referred to as #1 and #2). Additionally, two skin swabs and two tongue swabs were collected for each infant (see Fig. 1 for detailed information about timing of skin and oral swabs). For the skin and mouth, sample choice was based on the availability of sufficient DNA and, where more than two samples were available, to span the longest time period. In total, 183 gigabase-pairs of Illumina shotgun DNA sequencing were generated for 45 samples (Supplemental Table S2).

Historically, assembly-based metagenomics of the skin and mouth has been hampered by human DNA contamination (Liu et al. 2012; Tsai et al. 2016). While all but one skin or mouth sample consisted of >50% human DNA (range 34.2%–93.8%), the deep sequencing effort ensured that all samples had enough reads from microbial genomes for successful de novo genome reconstruction. Overall, an average of 98.7% (Infant 1) and 95.0% (Infant 2) of nonhuman reads could be assigned to assembled genomes (in some cases, by mapping to genomes reconstructed from another sample) (Supplemental Table S3).

Using ggKbase (Raveh-Sadka et al. 2015), we manually binned assembled DNA sequences to genome bins based on G+C content, coverage, and phylogenetic profile and subsequently used sequencing coverage patterns for binned scaffolds to verify the bins (Raveh-Sadka et al. 2015). For the bin refinement step, the clustering of fragments was analyzed using emergent self-organizing maps (ESOMs). Since identical genomes were assembled from different samples, the genomes were de-replicated based on similar average nucleotide identity (ANI), and the best genome for each strain was selected for downstream analyses. Across all three body sites for Infant 1, we recovered nine near-complete bacterial genomes and three partial genomes (all partial genomes were from the skin data sets). From Infant 2, we recovered 11 near-complete bacterial genomes and three partial genomes (all partial genomes were from the mouth). The community composition of all samples from all body sites and both infants are presented in Figure 1. It is interesting that the first two gut samples for Infant 2 that were collected during the antibiotic treatment course were dominated by *Streptococcus agalactiae*. The placenta showed heavy growth of *S. agalactiae* (group B streptococcus), yet, as noted above, the infant's blood cultures were negative.

**Figure 2.**
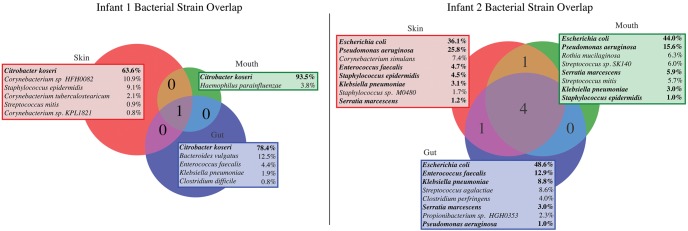
Identical bacterial strains colonize multiple body sites of premature infants. Microbes were considered colonists of a body site if they make up >1% of a community. All colonists of each site are shown, along with the total percentage of the community they make up across all sampling events. Colonists of multiple sites are shown in bold.

The overlap in community composition between body sites is shown in Figure 2. Strains were considered identical if they had over 99.9% whole-genome ANI and were considered colonizers of a body site if they accounted for >1% of reads in any sample from the site (Supplemental Table S4). Despite obvious differences in habitat characteristics, we identified some identical strain populations in all three sampled body sites for both infants. Infant 1 body sites were heavily colonized by *Citrobacter koseri*, which comprised over 60% of all three communities. Six strains were colonists of more than one body site of Infant 2: *Escherichia coli*, *Pseudomonas aeruginosa*, *Klebsiella pneumoniae*, and *Serratia marcescens* colonized all three body sites, *Enterococcus faecalis* colonized the mouth and skin, and *Staphylococcus epidermidis* colonized the mouth and gut. Interestingly *E. coli,* which is traditionally thought of as a gut colonist (Tenaillon et al. 2010), accounted for the highest portion of Infant 2 reads at all three body sites. The infants were housed in different NICU rooms ∼3 mo apart, and no bacterial strains were shared between infants.

We also tested for the presence of organisms in multiple body sites at low abundance (<1% of the community) and, to the extent possible, evaluated the time periods in which specific strains appeared at these sites (for details, see Methods). For Infant 1, *Enterococcus faecalis* had appeared in the skin and gut by the time of collection of the first samples, persisted in the gut, and was present in both habitats at the later time point when samples from both body sites were collected (day of life [DOL] 23). A *Haemophilus parainfluenzae* population was not detected in the first collected samples from all body sites but was present in the gut on DOL 21 and had appeared in the mouth and skin 2 d later. For Infant 2, *Staphylococcus epidermidis* and *E. coli* were present in all body sites at most time points. *Pseudomonas aeruginosa* and *Klebsiella pneumoniae* were undetectable in gut samples collected during antibiotic administration (Fig. 1; Supplemental Table S3) but appeared in all three body sites the day after cessation of antibiotics (DOL 7). *Staphylococcus sp.* M0480 was present by the time the first skin (DOL 12) and mouth (DOL 14) samples were collected but was undetectable in the mouth at the second time point. *E. faecalis* was present in the first-sampled gut and mouth communities (and persisted there), was absent in the skin on DOL 12, but had appeared there by DOL 22. *Serratia marcescens* was a relatively late colonist, appearing first in the gut on DOL 19 and was present in all three body sites at later time points (Supplemental Table S3). In general, strains became detectable at all three body sites at around the same day of life, with the exception of *E. faecalis* in Infant 2, which persisted in the gut and mouth for over a week before being detected in the skin.

### Growth rates are different across body sites

**Figure 3.**
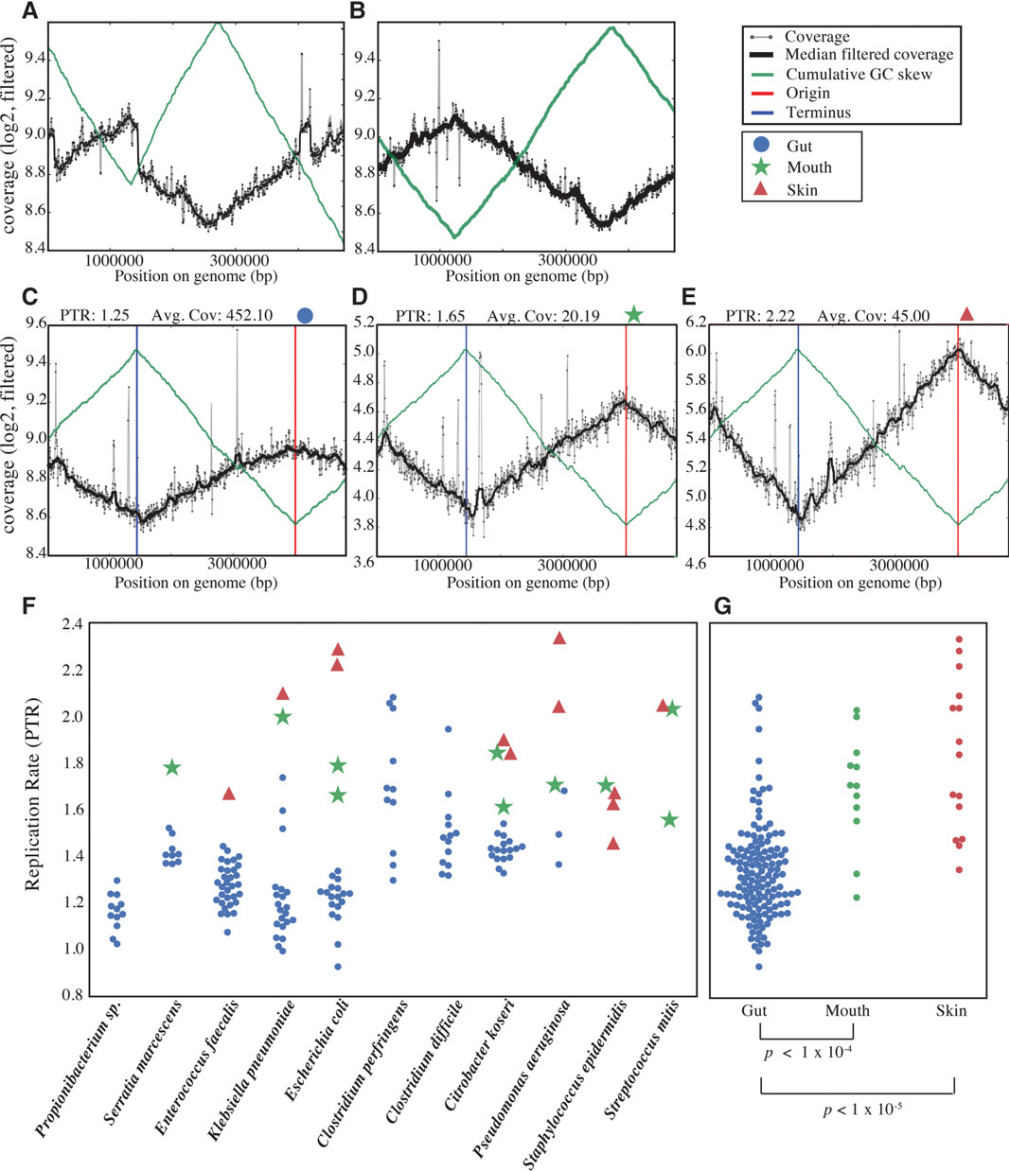
In situ bacterial growth rates are faster in the mouth and skin than the gut. (*A*,*B*) Cumulative GC-skew (green line) and coverage (black line) for our reconstructed *Citrobacter koseri* genome aligned to the RefSeq genome *C. koseri* strain ATCC BAA-895 (*A*) and another reference genome (*C. koseri* strain FDAARGOS_86) (*B*). Based on the irregularity of trends in the *C. koseri* strain ATCC BAA-895 plot, we conclude that this genome was improperly assembled at the rRNA operons. Thus, we used *C. koseri* strain FDAARGOS_86 for ordering and orienting our genome fragments. The ability to uncover assembly errors by inspection of PTR plots underlines the value of these displays. (*C*–*E*) Cumulative GC-skew and coverage of our ordered and oriented *Escherichia coli* genome from Infant 2 using reads mapped from a gut sample (*C*), mouth sample (*D*), and skin sample (*E*). Inspection of the PTR plots ensures that the origin and terminus are determined properly. (*F*) Aggregate of all peak-to-trough ratio (PTR) measurements for each bacterial species for which at least three measurements were available. In all cases where measurements are available for the same strain growing in multiple body sites, growth is slowest in the gut. (*G*) Direct comparison of all growth rate measurements for each body site. *P*-values for Mann-Whitney *U* test between body sites are shown *below*.

Recently, it was shown that accurate growth rates of microbial strains in their natural environment can be determined by measuring the ratio of the coverage of DNA at the origin and terminus of replication (Korem et al. 2015). However, this method requires complete closed circular genomes (which are rarely acquired from metagenomic studies) in order to locate the origin and terminus. We were able to circumvent that requirement by orienting each strain assembly (median number of contigs 67.5, range 12–1460) to a representative isolate genome in order to determine the order and orientation of the contigs. When available, we used multiple isolate genomes to confirm the best assembly, as some genomes in the RefSeq database were found to be incorrectly assembled around rRNA operons (Fig. 3A,B). The peak-to-trough ratio (origin to terminus coverage ratio) was then determined by mapping reads to the oriented assembly (see Methods).

Surprisingly, in all cases for which we could determine growth rates for the same strain from multiple body sites, growth was faster in the skin and mouth than in the gut (Fig. 3). When all growth rates were analyzed, microbes in the skin and mouth had significantly higher growth rates than microbes in the gut (*P* < 0.00001), whereas strains in the skin and mouth did not differ significantly in their growth rates (*P* = 0.12; Mann-Whitney *U* test). The strains exhibiting the fastest growth in the skin, mouth, and gut were *P. aeruginosa*, *Streptococcus mitis*, and *Clostridium perfringens*, respectively.

**Figure 4.**
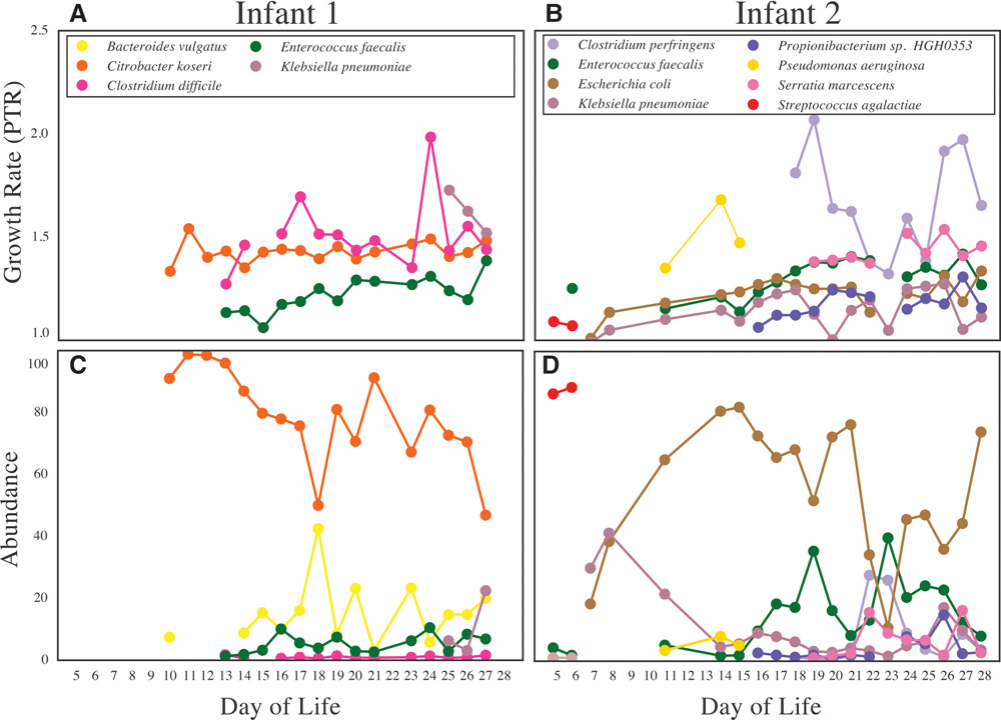
Growth rates (determined by PTR) often do not predict changes in relative abundance of a population in subsequent samples. Growth rate measurements for all gut colonists are shown in *A* and *B*. Corresponding relative abundance information is shown in *C* and *D*. The lack of correspondence between increased PTR in one sample and increased relative abundance in the next sample could be due either to the transient nature of growth spurts or to the fact that cell death is not accounted for in this analysis.

In general, growth rates measured for strains in the gut increased with increasing infant age, consistent with population recovery after early antibiotic treatment (Spearman rank correlation, *R* = 0.30, *P* = 0.0005, *n* = 135) (Fig. 4). In Infant 2, *S. agalactiae*, also known as group B Streptococcus, accounted for over 80% of the microbial community during antibiotic treatment in Infant 2 for a presumed group B Streptococcus infection. Despite being abundant, the organism exhibited a low and decreasing growth rate during the treatment period. Several microbial strains exhibited sharp changes in growth rates over the first month of life (*Clostridium difficile, C. perfringens, P. aeruginosa*). However, these events did not coincide with medical events indicated by the clinical metadata and were probably short lived, as they did not always lead to changes in relative abundance of these strains in the next-collected sample.

### Microdiversity

We classified strains colonizing multiple body sites as the same based on >99.9% genome-wide ANI, but this analysis is insensitive to very small-scale differences that could be used to document subpopulation dynamics and constrain inoculum diversity. Thus, we performed high-resolution analyses of *Citrobacter koseri,* the strain with the highest coverage in all body sites of Infant 1*.* The 4.66-Mbp genome was initially assembled de novo into 27 scaffolds. By reference to an isolate genome, we confirmed potential joins supported by sequence overlaps and identified gap-filling reads so that these scaffolds could be reconstructed into a complete circular genome.

**Figure 5.**
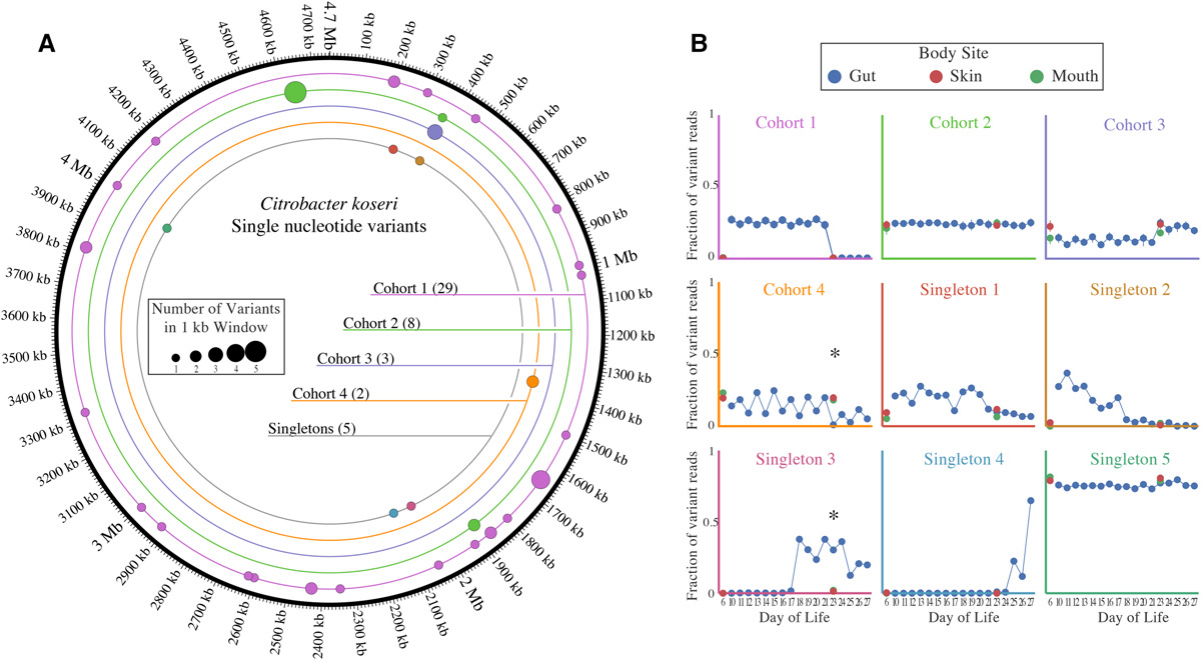
Subpopulations exist within colonizing *C. koseri* populations. (*A*) Single nucleotide variants were identified by mapping reads from all Infant 1 samples to the draft genome of *C. koseri* recovered from Infant 1. The total number of variants in each cohort is listed in parentheses. (*B*) The frequency of each variant in each sample. Cohorts are plotted as the average of all variant frequencies, with error bars representing standard deviation of the mean. Asterisks represent cases where the frequency of variants is statistically different between body sites (Fisher's exact *t*-test with Bonferroni correction, *P* < 0.01).

Single nucleotide polymorphisms were identified by mapping reads from each body site to the circularized *C. koseri* genome. No fixed mutations distinguished populations colonizing the skin, mouth, and gut. However, 47 polymorphic sites with a minimum frequency of 0.2 were identified along the genome. Of these, 21 occurred in intergenic regions and five within the coding region of *yadA*, a gene encoding adhesins with known pathogenic alleles (El Tahir and Skurnik 2001). Using hierarchical clustering, 10 cohorts of polymorphisms with similar variations in frequency over the sample series were identified, five of which had only one member (referred to as “singletons”) (Fig. 5). Each cohort is inferred to represent a strain subpopulation. Particularly interesting is cohort 1, which underwent a dramatic purge event around DOL 23, and singleton 3, which rapidly rose above detection level on DOL 17.

To evaluate differences in the populations across body sites, we focused on DOL 23, where gut, skin, and mouth samples are all available for Infant 1. Three variant positions had significantly different fractions of polymorphisms between the gut and both the skin and mouth (Fisher's exact test with Bonferroni correction, α = 0.01). These three positions make up the entirety of cohort 4 and singleton 3. Singleton 3 rises in abundance to comprise ∼40% of the gut population on DOL 17 but is only ever detected at ∼2% of the mouth and skin populations (Fig. 5B). There were no genomic positions with significantly different polymorphism levels between the mouth and skin on either DOL 6 or DOL 23. The detection of differences in subpopulation frequencies between body sites shows that there is limited gene flow between body sites and may indicate the start of in situ diversification.

### CRISPR and phage

In addition to the bacterial genomes referenced above, our assembly-based metagenomic pipeline resulted in the recovery of 21 bacteriophage genomes and 18 plasmid genomes. An average of 2.1% of reads from all samples mapped to bacteriophage genomes, and the most significant bacteriophage bloom occurred in the gut of Infant 2 on DOL 7 (immediately following cessation of antibiotic administration) (Fig. 1). Three bacteriophage genomes alone accounted for ∼50% of the DNA sequenced during this bloom: *K. pneumoniae* phage A (7.9% of community; 12,700× coverage), *K. pneumoniae* phage B (39.1% of community; 19,400× coverage), and *E. coli* phage A (2.7% of community; 4100× coverage). A second bloom of *K. pneumoniae* phage B and *E. coli* phage A also occurred in the same environment on DOL 24. Interestingly, although the bacteriophages’ abundance in the second bloom was only a fraction of their abundance in the first bloom, both blooms dramatically shifted the microbial community composition (Supplemental Fig. S1). Overall, however, most phage, plasmid, and host population abundance patterns were highly correlated (partly due to integration). Consequently, bacterial distribution patterns across the three body sites generally predicted patterns for the associated phage and plasmids (Supplemental Fig. S2). For circularly recovered phage and plasmids (inferred to be nonintegrated), we also found GC skew and coverage patterns that could be indicative of DNA replication style (Supplemental Fig. S3). Unfortunately, no phage or plasmids with these coverage patterns were detected at multiple time-points, so the consistency of this pattern could not be evaluated. However, such analyses could possibly be used to elucidate plasmid and phage replication regulation and growth rate in future studies.

CRISPR-Cas loci confer bacterial phage resistance (Horvath and Barrangou 2010). Because CRISPR spacers are added uni-directionally, the loci provide a record of population history (Sun et al. 2016). We identified CRISPR arrays in 50% of bacterial genomes in Infant 1 and 43% of bacterial genomes in Infant 2, with a total of 111 and 182 unique spacer sequences, respectively (Supplemental Table S5). However, we found only four spacer targets in the samples from the infant from which the spacers were recovered, even using relaxed (1 mismatch allowed) search parameters. Thus, we broadened our search to include previously published gut metagenomes from the same NICU as this study (Raveh-Sadka et al. 2015, 2016) and the current NCBI database. This revealed 26 and 16 spacers/protospacer matches, respectively.

**Figure 6.**
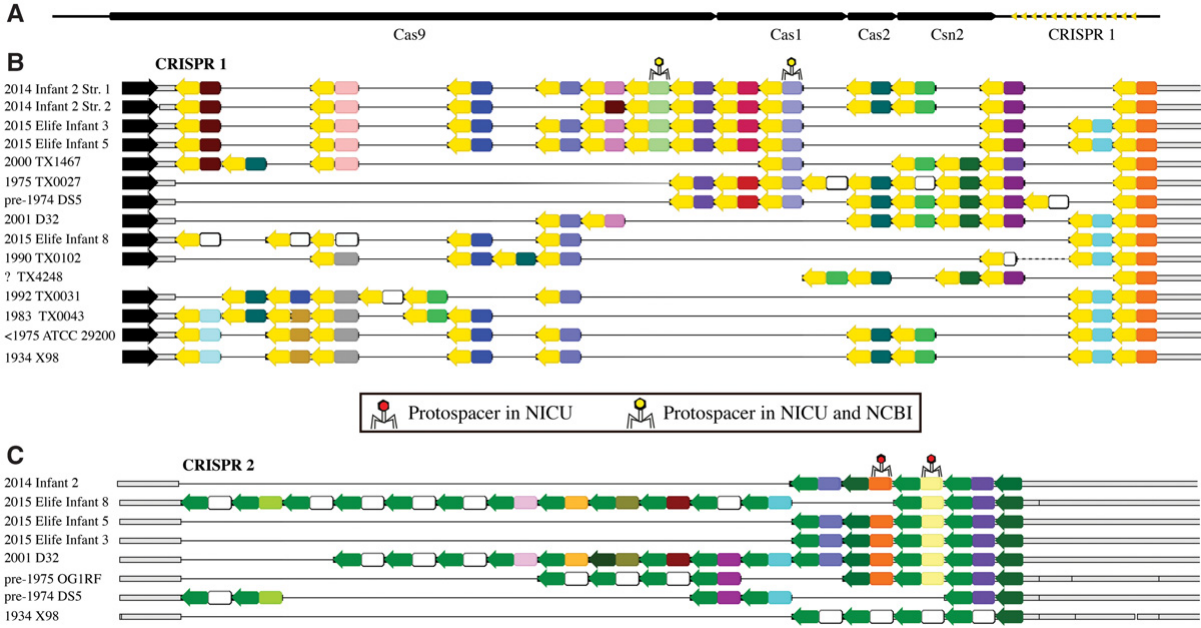
CRISPR spacers are maintained over decades in *Enterococcus faecalis*. (*A*) Genomic organization of CRISPR-Cas array #1. (*B*) Alignments of array #1 and (*C*) array #2 from *E. faecalis* from Infant 2 of this study compared to arrays reconstructed from publicly available genomes for isolates. The year of isolation of all *E. faecalis* isolates is provided to the extent possible. Infants marked “Elife” are those from a previous publication from the same NICU (Raveh-Sadka et al. 2015). Arrows represent repeats and colors represent spacers; identical colors symbolize identical spacers, whereas white spacers are unique. Phage symbols represent spacers with a protospacer match (max 1 mismatch) in a sequence assembled from infants in the same NICU as this study (red), and spacers with matches in both the same NICU and a separate genome in NCBI (yellow).

**Figure 7.**
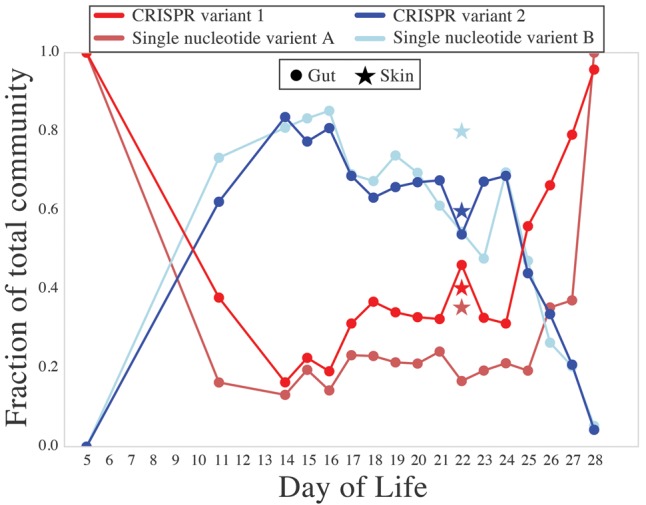
*E. faecalis* CRISPR variants change frequency within the *E. faecalis* population over the colonization period for Infant 2. The relative abundances of each CRISPR variant (diagrammed in Fig. 6) are shown. Additionally, two single nucleotide variants within *E. faecalis* correlated significantly with CRISPR variant frequencies (Pearson correlation with Bonferroni correction, *P* < 0.01). Both variants are located in the coding regions of hypothetical proteins.

No changes in CRISPR spacer inventories over the study period of 28 d were found, but two coexisting *E. faecalis* populations were identified in Infant 2 based on distinct CRISPR spacer inventories. The variant A locus contained 13 spacers and the variant B locus contained 11 unique spacers, with one spacer appearing in the array twice (Fig. 6). The ratio of the two variants fluctuated over the study period, with variant B rising from 0% of the population on DOL 5 to over 60% during the DOL 12–24 period and declining to ∼5% by DOL 28. As the spacers that differentiate variants A and B had no protospacer matches, we sought other genomic features for which selection might explain the abundance changes. One polymorphism was significantly correlated with the variant A locus and another significantly correlated with the variant B locus (Pearson correlation with Bonferroni correction, α = 0.01) (Fig. 7). Both polymorphisms were single-nucleotide substitutions within the coding regions of separate hypothetical proteins. Bacteria with both CRISPR locus variants and the associated gene variants were detected in the skin on DOL 22 (the only sample from another body site with sufficient coverage for detection). CRISPR arrays reconstructed in this study were also compared to a number of previously isolated *E. faecalis* strains (Fig. 6; Palmer and Gilmore 2010). Surprisingly, we found remarkable similarity in spacer content (with no polymorphisms in the spacer sequences) for *E. faecalis* strains isolated as many as 82 yr apart. Additionally, we found that isolates from the 1970s still retain CRISPR that match the sequences of phage still found in the NICU today.

## Discussion

Twenty-six bacterial genomes, as well as 39 phage/plasmid genomes, were recovered from two premature infants. Given that 98.7% of all reads mapped to 12 reconstructed bacterial and 18 phage/plasmid genomes from Infant 1, and 95.0% to 14 bacterial and 21 phage/plasmid genomes from Infant 2, we conclude that the majority of the community was accounted for. This result confirms the overall low diversity of the early community when compared to full-term infants (Costello et al. 2013; Gibson et al. 2016; Ward et al. 2016)*.* Overlap of strains across body sites contributes to the low total diversity of these premature infant microbiomes compared to those of other infants.

For *C. koseri,* we confirmed the complete absence of any fixed mutations that would distinguish populations in the mouth, skin, and gut, allowing us to conclude that identical populations colonized all three body sites. However, colonizing populations are typically not clonal (Luo et al. 2015). Analysis of subpopulation microdiversity, needed, for example, to constrain inoculum diversity, is complicated because recruitment of reads from different taxa to homologous regions can cause miscalculations in commonly used variant-detecting programs (Wilm et al. 2012; Deatherage and Barrick 2014). In this study, *C. koseri* had sufficient coverage in all Infant 1 samples to reliably detect variant positions (average coverage 493, full range 73–769), and no other similar taxa colonized the infant concurrently (preventing erroneous read recruitment). This allowed us to identify seven early colonizing *C. koseri* subpopulations (present in at least 20% of the reads) defined by between one and 29 polymorphisms, likely reflecting inoculation by at least seven distinct cell genotypes. Microdiversity has been linked with taxon stability in other environments (Jaspers and Overmann 2004; Rodriguez-Brito et al. 2010; Erkus et al. 2013), so seemingly low microdiversity in infant-associated populations may contribute to the observed low community stability as body habitats change along with infant development (Costello et al. 2013).

Our approach can constrain the timing and directionality of colonization events. For both infants, the presence of the same strains in multiple body sites at the first sampling event may indicate an early widespread inoculation event from the same source. The presence of the same population in multiple body sites suggests the ability of body sites to act as strain reservoirs for one another in early life. For example, colonization of the gut and mouth of Infant 2 by *E. faecalis* was followed by dispersal to the skin. However, we identified a strain of *C. koseri* that appeared later in the gut colonization process, but this strain did not spread to other body sites. This result may indicate increasing body site specificity as infant age increases and would imply functional significance of single mutations. This deduction may be supported by the dramatic shifts in abundances of genotypically near-identical *E. faecalis*, which were likely due to single nucleotide polymorphisms, given that CRISPR spacers that otherwise distinguish the variants did not have targets in the same samples.

The lack of coexisting CRISPR spacer targets (yet presence of targets in other samples) is likely due to the phage immunity conferred by the CRISPR spacers. Conservation of *E. faecalis* CRISPR spacers over many decades without mutation (Fig. 6) suggests that they target mutation-resistant phage genome regions. Slow CRISPR evolution contrasts with the dynamic seen in other systems (Tyson and Banfield 2007; Pride et al. 2011) but is consistent with observations of conserved CRISPR arrays in other common enteric organisms (Touchon and Rocha 2010; Touchon et al. 2011).

To our knowledge, this study represents the first comparison of in situ bacterial growth rates of multiple body sites, and the comparison is especially powerful as the measurements were for identical strains in different environments. In all cases when growth was measured for the same strain in multiple body sites, growth was slowest in the gut (Fig. 3C). Several mechanisms could explain the difference in growth between body sites, including differences in (1) higher resource availability (including oxygen) on the skin and in the mouth compared to the gut, (2) higher levels of competition among microbes in the gut, or (3) host control through the innate and/or adaptive immune system (Donaldson et al. 2015).

Previous studies have described the gut microbiome of premature infants as relatively simple and prone to rapid changes in composition (Costello et al. 2013; Gibson et al. 2016; Ward et al. 2016). To our knowledge, this is the first study to investigate the body habitat range of individual genotypes and to compare microbial activity of the same populations across body sites. Colonization of the three studied body sites by the same populations may be due to overall low inoculum diversity in the highly cleaned NICU and limited human contact. Given the rapid measured growth rates, the premature infant skin and mouth appear to be desirable microbial habitats (Fig. 3). It remains to be seen whether similar observations hold true for full-term infants, how long features of the founding communities persist, and whether differences in community composition arising from prematurity have long-term health consequences.

## Methods

### Patient recruitment and sample collection

Fecal samples from two preterm infants hospitalized in the NICU in Magee-Womens Hospital of UPMC (Pittsburgh, PA) were collected as available over the first month of life. Both infants were of low gestational age (<30 wk), and Infant 2 was of extremely low birth weight (<1000 g). See Supplemental Table S1 for additional clinical information.

Fecal samples were spontaneously expelled and collected from diapers or acquired directly using an established perineal stimulation procedure (Morowitz et al. 2011). Skin and oral samples were obtained by a member of the study team using a BD BBL Culture Swab EZ. Oral swabs were collected by rolling the swab head 5–10 times over the dorsal surface of the tongue. If intubated, the sample was collected by swabbing any exposed surface of the tongue. Skin swabs were collected by first dipping the swab into a 0.5 mL aliquot of a sterile solution of 0.15 M NaCl and 0.1% Tween 20. The swab head was then rolled 5–10 times over the left anterior upper chest wall. Stool samples were placed promptly into −20°C storage and transferred to a −80°C freezer for long-term storage as soon as possible. Swab samples were placed promptly in a −80°C freezer for storage.

DNA was extracted using either the MO BIO PowerSoil DNA Isolation kit (single tube extractions) or PowerSoil-htp 96-Well DNA Isolation kit. DNA extracted from stool using the single tube format followed the protocol as previously described (Raveh-Sadka et al. 2016). For DNA extracted from feces with the 96-well kit, fecal samples were added to individual wells of the bead plate and stored overnight at −80°C. The next day, the Bead Solution and Solution C1 were added, and the plates were incubated at 65°C for 10 min. The plates were shaken on a Retsch Oscillating Mill MM400 with 96-well plate adaptors for 10 min at speed 20. The plates were rotated 180° and shaken again for 10 min at speed 20. All remaining steps followed the manufacturer's centrifugation protocol. For swab samples, the swab head was cut off directly into the wells of the bead plate and stored overnight at −80°C. The next day, the Bead Solution and Solution C1 were added, and the plates were incubated at 65°C for 10 min. The plates were shaken on a Retsch Oscillating Mill MM400 with 96-well plate adaptors for 5 min at speed 20. The plates were rotated 180° and shaken again for 5 min at speed 20. The Solution C2 and C3 steps were combined (200 µL of each added) to improve DNA yield. All remaining steps followed the manufacturer's centrifugation protocol.

### Metagenomic sequencing and assembly

Sample preparation and sequencing of skin and oral samples were performed at the University of Illinois at Urbana-Champaign sequencing facility, and sample preparation and sequencing of fecal samples were performed at the University of California at Berkeley Vincent J. Coates Genomics Sequencing Laboratory. Paired end reads of 160 bp with a combination of 1000- and 600-bp library insert sizes were sequenced using an Illumina HiSeq 2500 (Supplemental Table S2). Reads were trimmed with Sickle (https://github.com/najoshi/sickle). Reads that mapped to the human genome with Bowtie 2 (Langmead and Salzberg 2012) under default settings were discarded. An additional step of mapping with BBMap (Bushnell 2014) was performed on all projects with at least 10% of reads removed with Bowtie 2 mapping. See Supplemental Table S2 for depth of sequencing and levels of human contamination in each sample.

Reads were assembled using idba_ud (Peng et al. 2012) under default settings. Resulting scaffolds >1 kb in length were annotated using Prodigal (Hyatt et al. 2010) to predict open reading frames using default metagenomic settings. Annotated protein sequences were searched against KEGG (Kanehisa et al. 2014), UniReff100 (Suzek et al. 2007), and UniProt databases using USEARCH (Edgar 2010). All matches with bit scores greater than 60 were saved, and reciprocal best hits with a bit score greater than 300 were also cataloged. We identified rRNA sequences using Infernal (Nawrocki and Eddy 2013) by searching against databases from the SSU-Align package (Nawrocki 2009) and tRNAs using tRNAscan_SE (Lowe and Eddy 1997).

Genome binning was carried out using the online interface within ggKbase as described previously (Raveh-Sadka et al. 2015; http://ggkbase.berkeley.edu/). This method takes into account phylogenetic profile, GC content, and coverage information. Bins were refined based on differential coverage implemented using time-series emergent self-organizing maps as described previously (Sharon et al. 2013). The completeness of bacterial bins was evaluated based on the presence or absence of single-copy genes (Raes et al. 2007; Raveh-Sadka et al. 2015). Phage sequences were identified based on the presence of typical phage genes such as capsid, terminase, and tail-fiber and as distinct clusters in ESOMs; phage-host relationships were inferred based on phylogeny of annotated proteins and abundance patterns.

### Genome recovery

All genome bins from all samples were pooled based on (1) the infant the genome was recovered from, and (2) the genome's identity as either of phage/plasmid origin or of bacterial origin. Genomes within each pool were then compared in a pairwise fashion based on ANIm (Richter and Rosselló-Móra 2009). For all clusters of genomes with high ANI values among members, a representative genome was chosen based on the highest total bin length, lowest scaffold fragmentation, and most complete complement of single-copy genes. Ambiguous genome clusters were visualized using Mauve alignments in Geneious (Kearse et al. 2012) to decide whether genomes could be included in the cluster.

Next, for each infant, ANIm was determined for all representative bacterial bins and all representative phage/plasmid bins together. The resulting ANI matrix was manually curated to resolve cases of overlap between the two genome sets. Most cases of overlap were of bacterial genomes containing prophage that were also represented in the phage list. These were resolved by removal of the prophage scaffold from the bacterial bin. The final genome list for each infant was verified by mapping reads from each project to the genomes to confirm strong coherence between the reads and the genomes, as well as to verify the completeness of the list based on total percentage of mapped project reads. Read-mapping data are available in Supplemental Table S3, and the final genome list is available in Supplemental Table S6.

### Sample profiling

Reads from all samples were mapped to the corresponding infant's genome list generated above. SAMtools (Li et al. 2009) was used to convert mapping files (.sam) to mpilup format, and calculate_breadth.py and pileup_profile.py were used to determine the depth of coverage, breadth of coverage, and average nucleotide identify of each genome in each project. As SAMtools has an implicit coverage limit of 8,000×, coverage values from calculate_breadth.py were used. The results of both scripts were manually combined and are available in Supplemental Table S7.

A strain was considered a “colonist” of a body site if at least 1% of the reads from at least one sample from at least one body site mapped to the recovered genome. We chose to define colonization in this way to be consistent with previous studies of infant colonization (Ward et al. 2016) and because using a coverage-based threshold would have biased against samples with less sequencing reads (Supplemental Fig. S4). The same analysis was performed on all phage and plasmid sequences, using 99% breadth to define carriage (Supplemental Fig. S2). To identify when strains below the 1% threshold first appeared in body sites, we normalized to account for different sampling depths by using a read percentage cutoff which corresponds to 0.1× coverage of the most shallowly sampled data set (see Jupyter notebook, CallingColonisits, for details).

### ANI calculation from metagenomic reads

Metagenomic reads from each sample were mapped to the genome list described previously, and nucleotide variants between reads and genomes were determined using pileup_profile.py. See the data availability section for full source code. Briefly, the script calculates ANI by masking regions of DNA near the ends of scaffolds, in conserved regions (tRNAs and rRNAs), or of insufficient coverage, locating all base pairs along the unmasked genome in which at least 80% of reads conflict with the reference genome, and calculating consensus ANI as (1−[# variant positions/unmasked genome length]). As shown in Supplemental Table S3, the number of SNPs found using this method was extremely low (average 34.2), with an average consensus ANI of 99.998%. We attempted to reduce the number of erroneously called SNPs and found that some appear to represent errors made during the process of metagenomic assembly (generation of the reference sequence) or unmasked regions of high sequence conservation (which recruit reads from other genomes) rather than real biological differences. Given this, and the extremely high reference ANI between strains on different body sites, we defined the strains as identical if they met the criterion of >99.9% consensus ANI.

### Growth rate determination

To attain growth rates for the incomplete genomes recovered in this study, genome fragments were ordered and oriented to previously isolated reference genomes and the peak-to-trough coverage ratio was determined using bPTR.py based on the method previously described (Korem et al. 2015; Brown et al. 2016) (Supplemental Table S8). Circular reference genomes of the same species as draft genomes from this study were downloaded from NCBI GenBank. The expected form of the cumulative GC skew of genomes (Grigoriev 1998) was manually verified using the program gc_skew.py, and genomes with aberrant patterns were discarded. The ANI of each draft genome to all reference genomes was determined using the previously described ANIm method, and the reference genome with the highest ANI was chosen. Draft genome fragments were aligned to the reference genome using BLAST (Altschul et al. 1990), and any fragment with <20% alignment coverage was discarded. The remaining draft sequence fragments were then aligned to the reference genome using progressive Mauve (Rissman et al. 2009) (java -Xmx500m -cp Mauve.jar org.gel.mauve.contigs.ContigOrderer), resulting in an ordered and oriented draft “core genome.” All core genomes were verified by manual inspection of the cumulative GC skew and genome coverage plots generated by the script (Supplemental Figs. S5, S6). Projects with aberrant plots or coverage below 5× were excluded from analysis.

### Microdiversity of *C. koseri*

To identify differences between the reads of specific data sets relative to reconstructed genomes (described above), VarScan (Koboldt et al. 2009) was run on all .pileup files using the pileup2cns command with the flag -min-coverage = 3. The frequency of each single nucleotide variant was tabulated for all samples using the script polymorpher2.py (Supplemental Table S9). Variants were then filtered based on a number of criteria and clustered based on changing relative frequency. Briefly, variants were required to have a minimum of 10× coverage in all samples, over 0.2 frequency in at least two samples, not be defined by polymorphisms present in conserved regions (rRNAs, tRNAs) (Supplemental Table S9), and pass an auto-correlation threshold. Clustering of variants was done using the Scipy hierarchical clustering package, with the cutoff threshold of 0.275 decided based on manual inspection of the resulting clusters. Full source code of analysis performed is available in the Jupyter notebook, CitroK_microdiversity (Supplemental Material).

### CRISPR analysis

CRISPR arrays were identified in bacterial draft genomes using the program CRISPRFinder (Grissa et al. 2007). CRISPR spacer targets (protospacers) were identified by searching spacers for full-length BLAST hits in a sequence database, followed by filtering our results that also included full-length matches to CRISPR repeats (to remove instances of the CRISPR array itself). In addition to the assemblies of this study, we also searched for protospacers in previously published studies from the same NICU (Raveh-Sadka et al. 2015, 2016) and the NCBI nt database (accessed January 2016). DNA fragments with identifiable CRISPR arrays were excluded from the protospacer search. The alignments of *E. faecalis* CRISPR arrays were manually curated within Geneious (Kearse et al. 2012). The ratio of variant arrays was determined by comparing the number of reads that mapped (using Bowtie 2 default settings) to unique regions of both arrays. Mutations elsewhere in the genome that varied in frequency with CRISPR variants were identified based on a significant Pearson correlation. Source code is available in the Jupyter notebook, *E. faecalis* microdiversity (Supplemental Material).

## Data access

The raw metagenomic reads from this study have been submitted to the NCBI Sequence Read Archive (SRA; http://www.ncbi.nlm.nih.gov/sra) under accession number SRP077514. The curated bacterial genomes from this study have been submitted to the NCBI BioProject (https://www.ncbi.nlm.nih.gov/bioproject/) under accession number PRJNA327106. Custom scripts used to analyze the data are available in the Supplemental Material and GitHub repositories https://github.com/banfieldlab/mattolm-public-scripts and https://github.com/christophertbrown/iRep. The source code and full methodological details of data analysis performed in Python are available in a number of Jupyter notebooks included in the Supplemental Material.

## Supplementary Material

Supplemental Material

## References

[OLMGR213256C1] Achtman M, Wagner M. 2008 Microbial diversity and the genetic nature of microbial species. Nat Rev Microbiol 6: 431–440. 18461076 10.1038/nrmicro1872

[OLMGR213256C2] Altschul SF, Gish W, Miller W, Myers EW, Lipman DJ. 1990 Basic local alignment search tool. J Mol Biol 215: 403–410. 2231712 10.1016/S0022-2836(05)80360-2

[OLMGR213256C3] Arrieta M-C, Stiemsma LT, Dimitriu PA, Thorson L, Russell S, Yurist-Doutsch S, Kuzeljevic B, Gold MJ, Britton HM, Lefebvre DL, 2015 Early infancy microbial and metabolic alterations affect risk of childhood asthma. Sci Transl Med 7: 307ra152. 10.1126/scitranslmed.aab2271 26424567

[OLMGR213256C4] Bäckhed F, Roswall J, Peng Y, Feng Q, Jia H, Kovatcheva-Datchary P, Li Y, Xia Y, Xie H, Zhong H, 2015 Dynamics and stabilization of the human gut microbiome during the first year of life. Cell Host Microbe 17: 690–703. 25974306 10.1016/j.chom.2015.04.004

[OLMGR213256C5] Brooks B, Firek BA, Miller CS, Sharon I, Thomas BC, Baker R, Morowitz MJ, Banfield JF. 2014 Microbes in the neonatal intensive care unit resemble those found in the gut of premature infants. Microbiome 2: 1. 24468033 10.1186/2049-2618-2-1 PMC4392516

[OLMGR213256C116] Brown CT, Olm MR, Thomas BC, Banfield JF. 2016 Measurement of bacterial replication rates in microbial communities. Nat Biotechnol 34: 1256–1263. 27819664 10.1038/nbt.3704 PMC5538567

[OLMGR213256C6] Browne HP, Forster SC, Anonye BO, Kumar N, Neville BA, Stares MD, Goulding D, Lawley TD. 2016 Culturing of ‘unculturable’ human microbiota reveals novel taxa and extensive sporulation. Nature 533: 543–546. 27144353 10.1038/nature17645 PMC4890681

[OLMGR213256C7] Bushnell B. 2014 BBMap: a fast, accurate, splice-aware aligner. Report Number: LBNL-7065E, Lawrence Berkeley National Laboratory, Berkeley, CA.

[OLMGR213256C8] Cahenzli J, Köller Y, Wyss M, Geuking MB, McCoy KD. 2013 Intestinal microbial diversity during early-life colonization shapes long-term IgE levels. Cell Host Microbe 14: 559–570. 24237701 10.1016/j.chom.2013.10.004 PMC4049278

[OLMGR213256C9] Cilieborg MS, Boye M, Sangild PT. 2012 Bacterial colonization and gut development in preterm neonates. Early Hum Dev 88: S41–S49. 22284985 10.1016/j.earlhumdev.2011.12.027

[OLMGR213256C10] Costello EK, Stagaman K, Dethlefsen L, Bohannan BJM, Relman DA. 2012 The application of ecological theory toward an understanding of the human microbiome. Science 336: 1255–1262. 22674335 10.1126/science.1224203 PMC4208626

[OLMGR213256C11] Costello EK, Carlisle EM, Bik EM, Morowitz MJ, Relman DA. 2013 Microbiome assembly across multiple body sites in low-birthweight infants. MBio 4: e00782–13. 24169577 10.1128/mBio.00782-13 PMC3809564

[OLMGR213256C12] Deatherage DE, Barrick JE. 2014 Identification of mutations in laboratory-evolved microbes from next-generation sequencing data using *breseq*. Methods Mol Biol 1151: 165–188. 24838886 10.1007/978-1-4939-0554-6_12 PMC4239701

[OLMGR213256C13] Ding T, Schloss PD. 2014 Dynamics and associations of microbial community types across the human body. Nature 509: 357–360. 24739969 10.1038/nature13178 PMC4139711

[OLMGR213256C14] Dominguez-Bello MG, Costello EK, Contreras M, Magris M, Hidalgo G, Fierer N, Knight R. 2010 Delivery mode shapes the acquisition and structure of the initial microbiota across multiple body habitats in newborns. Proc Natl Acad Sci 107: 11971–11975. 20566857 10.1073/pnas.1002601107 PMC2900693

[OLMGR213256C15] Dominguez-Bello MG, De Jesus-Laboy KM, Shen N, Cox LM, Amir A, Gonzalez A, Bokulich NA, Song SJ, Hoashi M, Rivera-Vinas JI, 2016 Partial restoration of the microbiota of cesarean-born infants via vaginal microbial transfer. Nat Med 22: 250–253. 26828196 10.1038/nm.4039 PMC5062956

[OLMGR213256C16] Donaldson GP, Lee SM, Mazmanian SK. 2015 Gut biogeography of the bacterial microbiota. Nat Rev Microbiol 14: 20–32. 26499895 10.1038/nrmicro3552 PMC4837114

[OLMGR213256C17] Edgar RC. 2010 Search and clustering orders of magnitude faster than BLAST. Bioinformatics 26: 2460–2461. 20709691 10.1093/bioinformatics/btq461

[OLMGR213256C18] El Tahir Y, Skurnik M. 2001 YadA, the multifaceted *Yersinia* adhesin. Int J Med Microbiol 291: 209–218. 11554561 10.1078/1438-4221-00119

[OLMGR213256C19] Erkus O, de Jager VC, Spus M, van Alen-Boerrigter IJ, van Rijswijck IM, Hazelwood L, Janssen PW, van Hijum SA, Kleerebezem M, Smid EJ. 2013 Multifactorial diversity sustains microbial community stability. ISME J 7: 2126–2136. 23823494 10.1038/ismej.2013.108 PMC3806261

[OLMGR213256C20] Faith JJ, Colombel J-F, Gordon JI. 2015 Identifying strains that contribute to complex diseases through the study of microbial inheritance. Proc Natl Acad Sci 112: 633–640. 25576328 10.1073/pnas.1418781112 PMC4311841

[OLMGR213256C21] Gibson MK, Wang B, Ahmadi S, Burnham C-AD, Tarr PI, Warner BB, Dantas G. 2016 Developmental dynamics of the preterm infant gut microbiota and antibiotic resistome. Nat Microbiol 1: 16024. 27572443 10.1038/nmicrobiol.2016.24 PMC5031140

[OLMGR213256C22] Grigoriev A. 1998 Analyzing genomes with cumulative skew diagrams. Nucleic Acids Res 26: 2286–2290. 9580676 10.1093/nar/26.10.2286 PMC147580

[OLMGR213256C23] Grissa I, Vergnaud G, Pourcel C. 2007 CRISPRFinder: a web tool to identify clustered regularly interspaced short palindromic repeats. Nucleic Acids Res 35: W52–W57. 17537822 10.1093/nar/gkm360 PMC1933234

[OLMGR213256C24] Horvath P, Barrangou R. 2010 CRISPR/Cas, the immune system of bacteria and archaea. Science 327: 167–170. 20056882 10.1126/science.1179555

[OLMGR213256C25] Hyatt D, Chen G-L, LoCascio PF, Land ML, Larimer FW, Hauser LJ. 2010 Prodigal: prokaryotic gene recognition and translation initiation site identification. BMC Bioinformatics 11: 119. 20211023 10.1186/1471-2105-11-119 PMC2848648

[OLMGR213256C26] Jaspers E, Overmann J. 2004 Ecological significance of microdiversity: Identical 16S rRNA gene sequences can be found in bacteria with highly divergent genomes and ecophysiologies. Appl Environ Microbiol 70: 4831–4839. 15294821 10.1128/AEM.70.8.4831-4839.2004 PMC492463

[OLMGR213256C28] Jovel J, Patterson J, Wang W, Hotte N, O'Keefe S, Mitchel T, Perry T, Kao D, Mason AL, Madsen KL, 2016 Characterization of the gut microbiome using 16S or shotgun metagenomics. Front Microbiol 7: 459. 27148170 10.3389/fmicb.2016.00459 PMC4837688

[OLMGR213256C29] Kanehisa M, Goto S, Sato Y, Kawashima M, Furumichi M, Tanabe M. 2014 Data, information, knowledge and principle: back to metabolism in KEGG. Nucleic Acids Res 42: D199–D205. 24214961 10.1093/nar/gkt1076 PMC3965122

[OLMGR213256C30] Karl DM. 1979 Measurement of microbial activity and growth in the ocean by rates of stable ribonucleic acid synthesis. Appl Environ Microbiol 38: 850–860. 16345461 10.1128/aem.38.5.850-860.1979 PMC243599

[OLMGR213256C31] Kearse M, Moir R, Wilson A, Stones-Havas S, Cheung M, Sturrock S, Buxton S, Cooper A, Markowitz S, Duran C, 2012 Geneious Basic: an integrated and extendable desktop software platform for the organization and analysis of sequence data. Bioinformatics 28: 1647–1649. 22543367 10.1093/bioinformatics/bts199 PMC3371832

[OLMGR213256C32] King KC, Brockhurst MA, Vasieva O, Paterson S, Betts A, Ford SA, Frost CL, Horsburgh MJ, Haldenby S, Hurst GD. 2016 Rapid evolution of microbe-mediated protection against pathogens in a worm host. ISME J 10: 1915–1924. 26978164 10.1038/ismej.2015.259 PMC5029159

[OLMGR213256C33] Koboldt DC, Chen K, Wylie T, Larson DE, McLellan MD, Mardis ER, Weinstock GM, Wilson RK, Ding L. 2009 VarScan: variant detection in massively parallel sequencing of individual and pooled samples. Bioinformatics 25: 2283–2285. 19542151 10.1093/bioinformatics/btp373 PMC2734323

[OLMGR213256C34] Korem T, Zeevi D, Suez J, Weinberger A, Avnit-Sagi T, Pompan-Lotan M, Matot E, Jona G, Harmelin A, Cohen N, 2015 Growth dynamics of gut microbiota in health and disease inferred from single metagenomic samples. Science 349: 1101–1106. 26229116 10.1126/science.aac4812 PMC5087275

[OLMGR213256C35] Lang GI, Rice DP, Hickman MJ, Sodergren E, Weinstock GM, Botstein D, Desai MM. 2013 Pervasive genetic hitchhiking and clonal interference in forty evolving yeast populations. Nature 500: 571–574. 23873039 10.1038/nature12344 PMC3758440

[OLMGR213256C36] Langmead B, Salzberg SL. 2012 Fast gapped-read alignment with Bowtie 2. Nat Methods 9: 357–359. 22388286 10.1038/nmeth.1923 PMC3322381

[OLMGR213256C37] Li H, Handsaker B, Wysoker A, Fennell T, Ruan J, Homer N, Marth G, Abecasis G, Durbin R. 2009 The Sequence Alignment/Map format and SAMtools. Bioinformatics 25: 2078–2079. 19505943 10.1093/bioinformatics/btp352 PMC2723002

[OLMGR213256C38] Liu B, Faller LL, Klitgord N, Mazumdar V, Ghodsi M, Sommer DD, Gibbons TR, Treangen TJ, Chang Y-C, Li S, 2012 Deep sequencing of the oral microbiome reveals signatures of periodontal disease. PLoS One 7: e37919. 22675498 10.1371/journal.pone.0037919 PMC3366996

[OLMGR213256C39] Lowe TM, Eddy SR. 1997 tRNAscan-SE: a program for improved detection of transfer RNA genes in genomic sequence. Nucleic Acids Res 25: 955–964. 9023104 10.1093/nar/25.5.955 PMC146525

[OLMGR213256C40] Luo C, Walk ST, Gordon DM, Feldgarden M, Tiedje JM, Konstantinidis KT. 2011 Genome sequencing of environmental *Escherichia coli* expands understanding of the ecology and speciation of the model bacterial species. Proc Natl Acad Sci 108: 7200–7205. 21482770 10.1073/pnas.1015622108 PMC3084108

[OLMGR213256C41] Luo C, Knight R, Siljander H, Knip M, Xavier RJ, Gevers D. 2015 ConStrains identifies microbial strains in metagenomic datasets. Nat Biotechnol 33: 1045–1052. 26344404 10.1038/nbt.3319 PMC4676274

[OLMGR213256C42] Morowitz MJ, Denef VJ, Costello EK, Thomas BC, Poroyko V, Relman DA, Banfield JF. 2011 Strain-resolved community genomic analysis of gut microbial colonization in a premature infant. Proc Natl Acad Sci 108: 1128–1133. 21191099 10.1073/pnas.1010992108 PMC3024690

[OLMGR213256C43] Mueller NT, Bakacs E, Combellick J, Grigoryan Z, Dominguez-Bello MG. 2015 The infant microbiome development: mom matters. Trends Mol Med 21: 109–117. 25578246 10.1016/j.molmed.2014.12.002 PMC4464665

[OLMGR213256C44] Nawrocki E. 2009 “Structural RNA homology search and alignment using covariance models.” PhD dissertation, Washington University, St. Louis, MO http://openscholarship.wustl.edu/etd/256/.

[OLMGR213256C45] Nawrocki EP, Eddy SR. 2013 Infernal 1.1: 100-fold faster RNA homology searches. Bioinformatics 29: 2933–2935. 24008419 10.1093/bioinformatics/btt509 PMC3810854

[OLMGR213256C46] Nocker A, Cheung C-Y, Camper AK. 2006 Comparison of propidium monoazide with ethidium monoazide for differentiation of live vs. dead bacteria by selective removal of DNA from dead cells. J Microbiol Methods 67: 310–320. 16753236 10.1016/j.mimet.2006.04.015

[OLMGR213256C47] Palmer KL, Gilmore MS. 2010 Multidrug-resistant *Enterococci* lack CRISPR-*cas*. MBio 1: e00227-10–e00227-19. 21060735 10.1128/mBio.00227-10 PMC2975353

[OLMGR213256C48] Peng Y, Leung HCM, Yiu SM, Chin FYL. 2012 IDBA-UD: a de novo assembler for single-cell and metagenomic sequencing data with highly uneven depth. Bioinformatics 28: 1420–1428. 22495754 10.1093/bioinformatics/bts174

[OLMGR213256C49] Pride DT, Sun CL, Salzman J, Rao N, Loomer P, Armitage GC, Banfield JF, Relman DA. 2011 Analysis of streptococcal CRISPRs from human saliva reveals substantial sequence diversity within and between subjects over time. Genome Res 21: 126–136. 21149389 10.1101/gr.111732.110 PMC3012920

[OLMGR213256C50] Prosser JI, Bohannan BJ, Curtis TP, Ellis RJ, Firestone MK, Freckleton RP, Green JL, Green LE, Killham K, Lennon JJ. 2007 The role of ecological theory in microbial ecology. Nat Rev Microbiol 5: 384–392. 17435792 10.1038/nrmicro1643

[OLMGR213256C51] Raes J, Korbel JO, Lercher MJ, Von Mering C, Bork P. 2007 Prediction of effective genome size in metagenomic samples. Genome Biol 8: 1. 10.1186/gb-2007-8-1-r10 PMC1839125 17224063

[OLMGR213256C52] Raveh-Sadka T, Thomas BC, Singh A, Firek B, Brooks B, Castelle CJ, Sharon I, Baker R, Good M, Morowitz MJ, 2015 Gut bacteria are rarely shared by co-hospitalized premature infants, regardless of necrotizing enterocolitis development. eLife 4: e05477. 10.7554/eLife.05477 PMC4384745 25735037

[OLMGR213256C53] Raveh-Sadka T, Firek B, Sharon I, Baker R, Brown CT, Thomas BC, Morowitz MJ, Banfield JF. 2016 Evidence for persistent and shared bacterial strains against a background of largely unique gut colonization in hospitalized premature infants. ISME J 10: 2817–2830. 27258951 10.1038/ismej.2016.83 PMC5148203

[OLMGR213256C54] Richter M, Rosselló-Móra R. 2009 Shifting the genomic gold standard for the prokaryotic species definition. Proc Natl Acad Sci 106: 19126–19131. 19855009 10.1073/pnas.0906412106 PMC2776425

[OLMGR213256C55] Rissman AI, Mau B, Biehl BS, Darling AE, Glasner JD, Perna NT. 2009 Reordering contigs of draft genomes using the Mauve Aligner. Bioinformatics 25: 2071–2073. 19515959 10.1093/bioinformatics/btp356 PMC2723005

[OLMGR213256C56] Rodriguez GG, Phipps D, Ishiguro K, Ridgway HF. 1992 Use of a fluorescent redox probe for direct visualization of actively respiring bacteria. Appl Environ Microbiol 58: 1801–1808. 1622256 10.1128/aem.58.6.1801-1808.1992 PMC195687

[OLMGR213256C57] Rodriguez-Brito B, Li L, Wegley L, Furlan M, Angly F, Breitbart M, Buchanan J, Desnues C, Dinsdale E, Edwards R, 2010 Viral and microbial community dynamics in four aquatic environments. ISME J 4: 739–751. 20147985 10.1038/ismej.2010.1

[OLMGR213256C58] Rosen MJ, Davison M, Bhaya D, Fisher DS. 2015 Fine-scale diversity and extensive recombination in a quasisexual bacterial population occupying a broad niche. Science 348: 1019–1023. 26023139 10.1126/science.aaa4456

[OLMGR213256C59] Salzberg SL, Yorke JA. 2005 Beware of mis-assembled genomes. Bioinformatics 21: 4320–4321. 16332717 10.1093/bioinformatics/bti769

[OLMGR213256C60] Sharon I, Morowitz MJ, Thomas BC, Costello EK, Relman DA, Banfield JF. 2013 Time series community genomics analysis reveals rapid shifts in bacterial species, strains, and phage during infant gut colonization. Genome Res 23: 111–120. 22936250 10.1101/gr.142315.112 PMC3530670

[OLMGR213256C61] Shin H, Pei Z, Martinez KA, Rivera-Vinas JI, Mendez K, Cavallin H, Dominguez-Bello MG. 2015 The first microbial environment of infants born by C-section: the operating room microbes. Microbiome 3: 59. 26620712 10.1186/s40168-015-0126-1 PMC4665759

[OLMGR213256C62] Sim K, Powell E, Shaw AG, McClure Z, Bangham M, Kroll JS. 2013 The neonatal gastrointestinal microbiota: the foundation of future health? Arch Dis Child Fetal Neonatal Ed 98: F362–F364. 23221466 10.1136/archdischild-2012-302872

[OLMGR213256C63] Snitkin ES, Zelazny AM, Thomas PJ, Stock F; NISC Comparative Sequencing Program Group, Henderson DK, Palmore TN, Segre JA. 2012 Tracking a hospital outbreak of carbapenem-resistant *Klebsiella pneumoniae* with whole-genome sequencing. Sci Transl Med 4: 148ra116. 10.1126/scitranslmed.3004129 PMC3521604 22914622

[OLMGR213256C64] Sun CL, Thomas BC, Barrangou R, Banfield JF. 2016 Metagenomic reconstructions of bacterial CRISPR loci constrain population histories. ISME J 10: 858–870. 26394009 10.1038/ismej.2015.162 PMC4796926

[OLMGR213256C65] Suzek BE, Huang H, McGarvey P, Mazumder R, Wu CH. 2007 UniRef: comprehensive and non-redundant UniProt reference clusters. Bioinformatics 23: 1282–1288. 17379688 10.1093/bioinformatics/btm098

[OLMGR213256C66] Tenaillon O, Skurnik D, Picard B, Denamur E. 2010 The population genetics of commensal *Escherichia coli*. Nat Rev Microbiol 8: 207–217. 20157339 10.1038/nrmicro2298

[OLMGR213256C67] Touchon M, Rocha EPC. 2010 The small, slow and specialized CRISPR and anti-CRISPR of *Escherichia* and *Salmonella*. PLoS One 5: e11126. 20559554 10.1371/journal.pone.0011126 PMC2886076

[OLMGR213256C68] Touchon M, Charpentier S, Clermont O, Rocha EPC, Denamur E, Branger C. 2011 CRISPR distribution within the *Escherichia coli* species is not suggestive of immunity-associated diversifying selection. J Bacteriol 193: 2460–2467. 21421763 10.1128/JB.01307-10 PMC3133152

[OLMGR213256C69] Tsai Y-C, Conlan S, Deming C; NISC Comparative Sequencing Program, Segre JA, Kong HH, Korlach J, Oh J. 2016 Resolving the complexity of human skin metagenomes using single-molecule sequencing. MBio 7: e01948–15. 26861018 10.1128/mBio.01948-15 PMC4752602

[OLMGR213256C70] Tu Q, He Z, Zhou J. 2014 Strain/species identification in metagenomes using genome-specific markers. Nucleic Acids Res 42: e67. 24523352 10.1093/nar/gku138 PMC4005670

[OLMGR213256C71] Tyson GW, Banfield JF. 2007 Rapidly evolving CRISPRs implicated in acquired resistance of microorganisms to viruses. Environ Microbiol 10: 200–207. 17894817 10.1111/j.1462-2920.2007.01444.x

[OLMGR213256C72] Ward DV, Scholz M, Zolfo M, Taft DH, Schibler KR, Tett A, Segata N, Morrow AL. 2016 Metagenomic sequencing with strain-level resolution implicates uropathogenic *E. coli* in necrotizing enterocolitis and mortality in preterm infants. Cell Rep 14: 2912–2924. 26997279 10.1016/j.celrep.2016.03.015 PMC4819403

[OLMGR213256C73] Wilm A, Aw PPK, Bertrand D, Yeo GHT, Ong SH, Wong CH, Khor CC, Petric R, Hibberd ML, Nagarajan N. 2012 LoFreq: a sequence-quality aware, ultra-sensitive variant caller for uncovering cell-population heterogeneity from high-throughput sequencing datasets. Nucleic Acids Res 40: 11189–11201. 23066108 10.1093/nar/gks918 PMC3526318

