# Supplementary material for: Identical bacterial populations colonize premature infant gut, skin, and oral microbiomes and exhibit different in situ growth rates
Source: Genome Res. 2017 Apr;27(4):601–12. doi: 10.1101/gr.213256.116 (PMC5378178; doi:10.1101/gr.213256.116)
Supplement: Supplemental Material [file supp_27_4_601__index.html]

Identical bacterial populations colonize premature infant gut, skin, and oral microbiomes and exhibit different in situ growth rates — Supplemental Material 

# Identical bacterial populations colonize premature infant gut, skin, and oral microbiomes and exhibit different in situ growth rates

## Supplemental Material

- Supplemental\_Fig\_S1.pdf
- Supplemental\_Fig\_S2.pdf
- Supplemental\_Fig\_S3.pdf
- Supplemental\_Fig\_S4.pdf
- Supplemental\_Fig\_S5.pdf
- Supplemental\_Fig\_S6.pdf
- Supplemental\_Table\_S1.xlsx
- Supplemental\_Table\_S2.xlsx
- Supplemental\_Table\_S3.csv
- Supplemental\_Table\_S4.xlsx
- Supplemental\_Table\_S5.xlsx
- Supplemental\_Table\_S6.xls
- Supplemental\_Table\_S7.xlsx
- Supplemental\_Table\_S8.xlsx
- Supplemental\_Table\_S9.xlsx
- Supplemental\_Pileup.py
- Supplemental\_calculate.py
- Supplemental\_polymor.py
- Supplemental\_Jupyter\_S1-5.zip
