# Supplementary material for: Identical bacterial populations colonize premature infant gut, skin, and oral microbiomes and exhibit different in situ growth rates
Source: Genome Res. 2017 Apr;27(4):601–12. doi: 10.1101/gr.213256.116 (PMC5378178; doi:10.1101/gr.213256.116)
Supplement: Supplemental Material [file supp_gr.213256.116_Supplemental_Fig_S1.pdf]

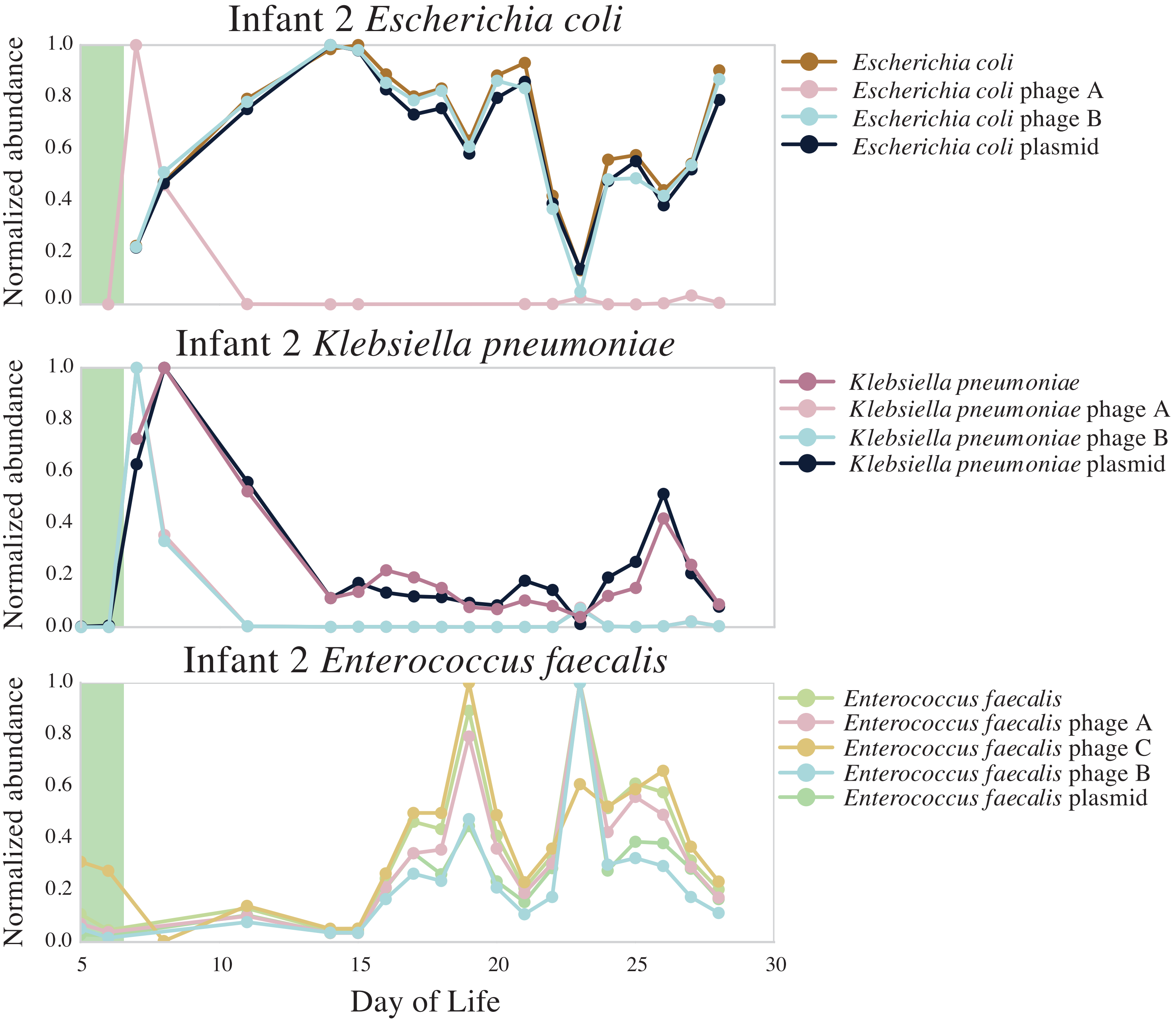

Figure S1 Mobile elements (phage/plasmid) and bacterial genomes exhibit similar changes in abundance over the first month of life. Shown are results for selected bacteria and their associated mobile elements. The abundance of each genome was normalized separately to the highest abundance of that genome in any gut sample. Green bar indicates the period of antibiotic administration. Most phage and plasmid genomes display similar abundance patterns as their hosts, although two phage bloom immediately following the cessation of antibiotic administration.
