# Supplementary material for: Identical bacterial populations colonize premature infant gut, skin, and oral microbiomes and exhibit different in situ growth rates
Source: Genome Res. 2017 Apr;27(4):601–12. doi: 10.1101/gr.213256.116 (PMC5378178; doi:10.1101/gr.213256.116)
Supplement: Supplemental Material [file supp_gr.213256.116_Supplemental_Fig_S2.pdf]

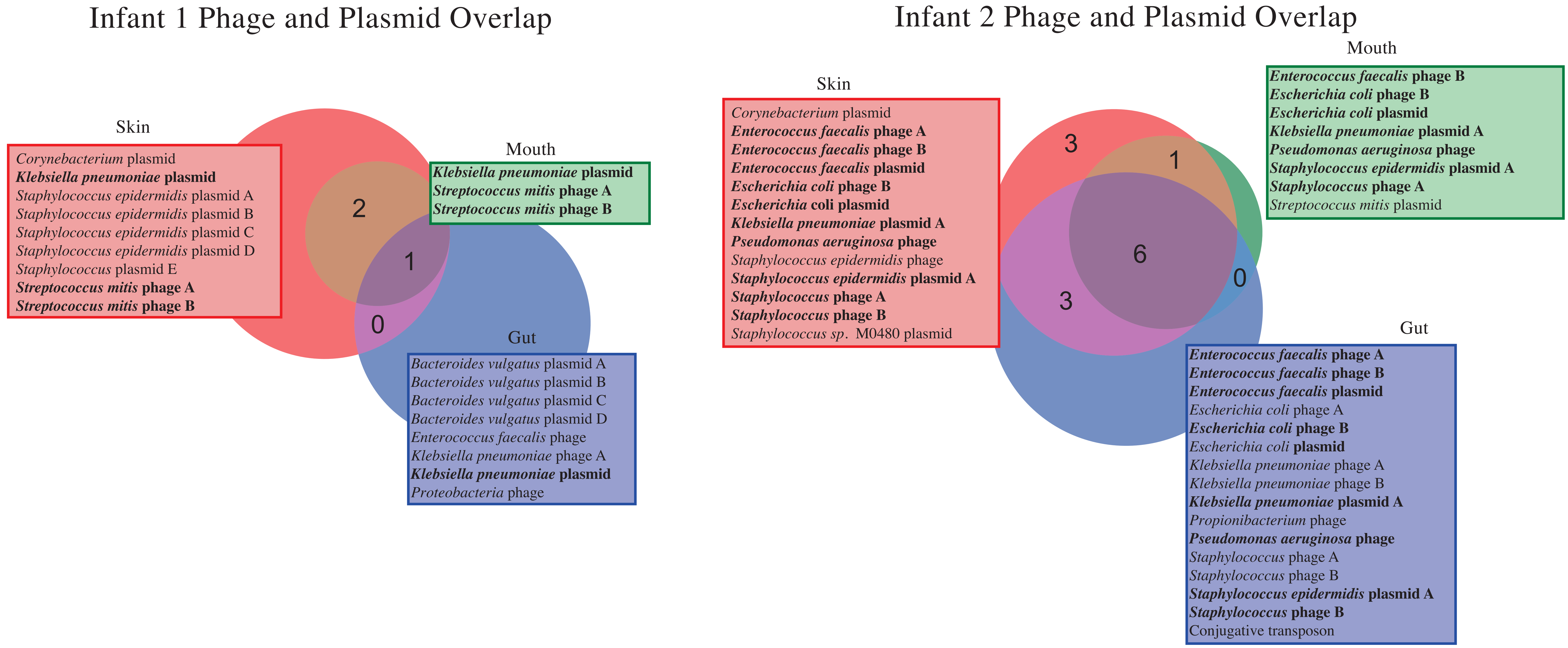

Figure S2 There is significant overlap of bacteriophage and plasmids across body sites. Phage and plasmids were included in the analysis if a genome reconstructed from one sample was covered across 99% of its length by reads from at least one sample from another body site. Genomes with names in bold are found in at least two body sites. In general, mobile element body-site sharing paralleled that of the presumed host bacteria.
