# Supplementary material for: Identical bacterial populations colonize premature infant gut, skin, and oral microbiomes and exhibit different in situ growth rates
Source: Genome Res. 2017 Apr;27(4):601–12. doi: 10.1101/gr.213256.116 (PMC5378178; doi:10.1101/gr.213256.116)
Supplement: Supplemental Material [file supp_gr.213256.116_Supplemental_Fig_S3.pdf]

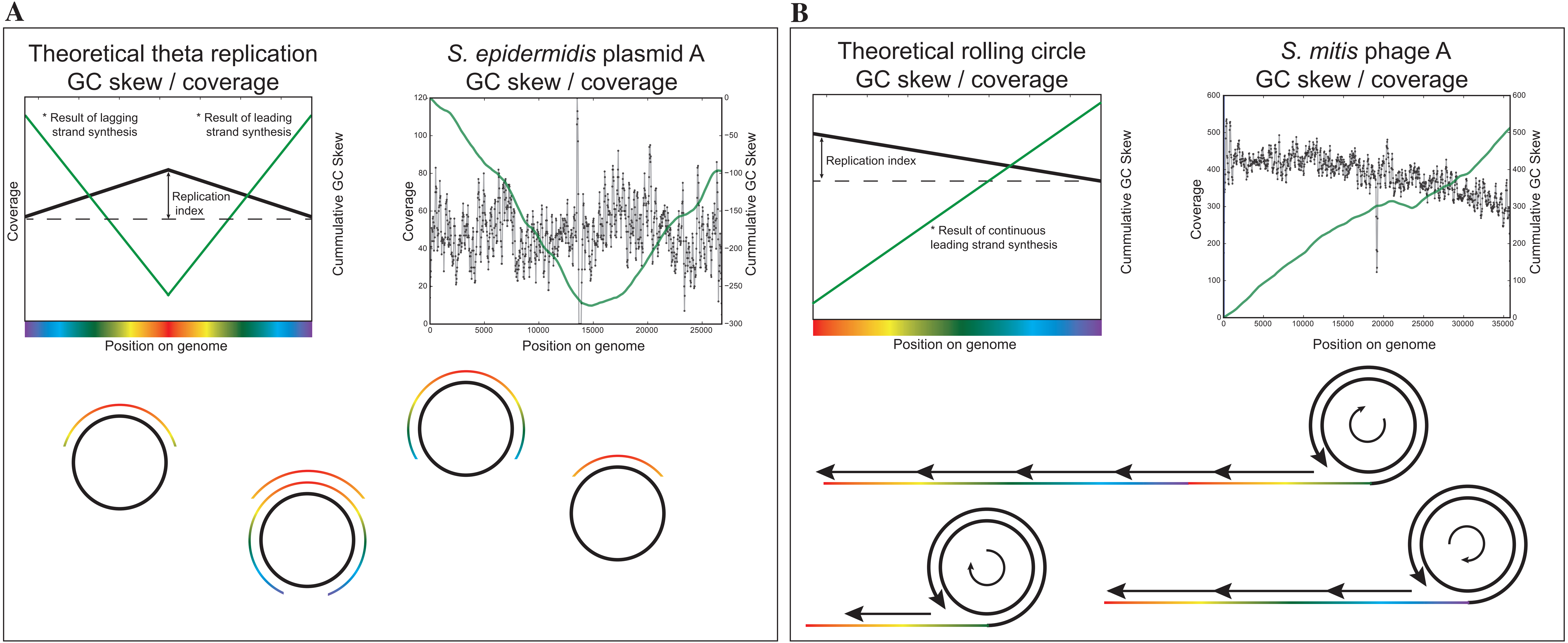

Figure S3 Cumulative GC Skew and coverage profiles of assembled circular pieces of DNA may be indicative of replication style. For both rolling circle replication (A) and theta replication (B), a schematic of the replication style is shown (bottom). Theoretical cumulative GC skew and coverage plots are illustrated (left) based on interpretation of the schematics, and are compared to actual cumulative GC skew and coverage plots of assembled pieces of circular DNA (right).
