# Supplementary material for: Identical bacterial populations colonize premature infant gut, skin, and oral microbiomes and exhibit different in situ growth rates
Source: Genome Res. 2017 Apr;27(4):601–12. doi: 10.1101/gr.213256.116 (PMC5378178; doi:10.1101/gr.213256.116)
Supplement: Supplemental Material [file supp_gr.213256.116_Supplemental_Fig_S4.pdf]

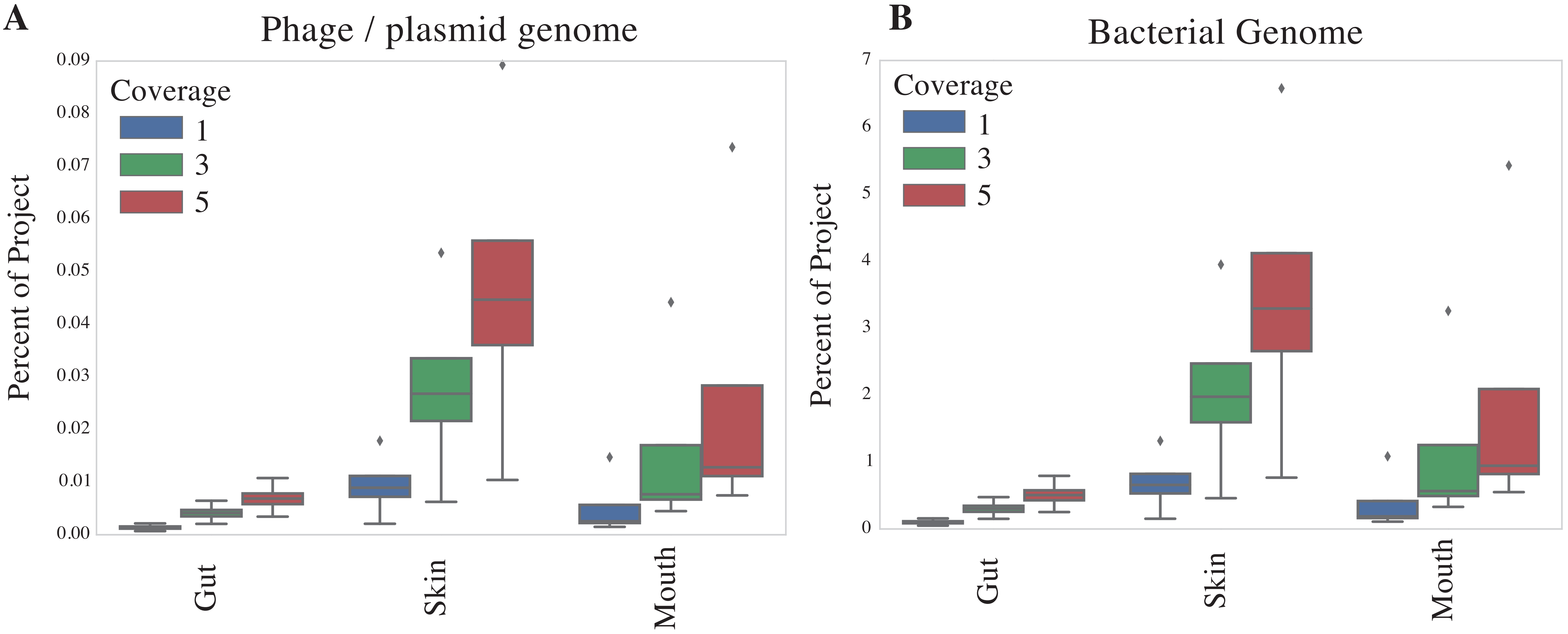

Figure S4 Body sites have different depths of sequencing, due largely to extensive human DNA contamination. For the average size phage/plasmid (a) (44 kb) and bacterial (b) (3.3 Mbp) genome, the percentage of project reads that correspond with different coverage levels is shown for all three body sites.
