# Supplementary material for: Identical bacterial populations colonize premature infant gut, skin, and oral microbiomes and exhibit different in situ growth rates
Source: Genome Res. 2017 Apr;27(4):601–12. doi: 10.1101/gr.213256.116 (PMC5378178; doi:10.1101/gr.213256.116)
Supplement: Supplemental Material [file supp_gr.213256.116_Supplemental_Fig_S6.pdf]

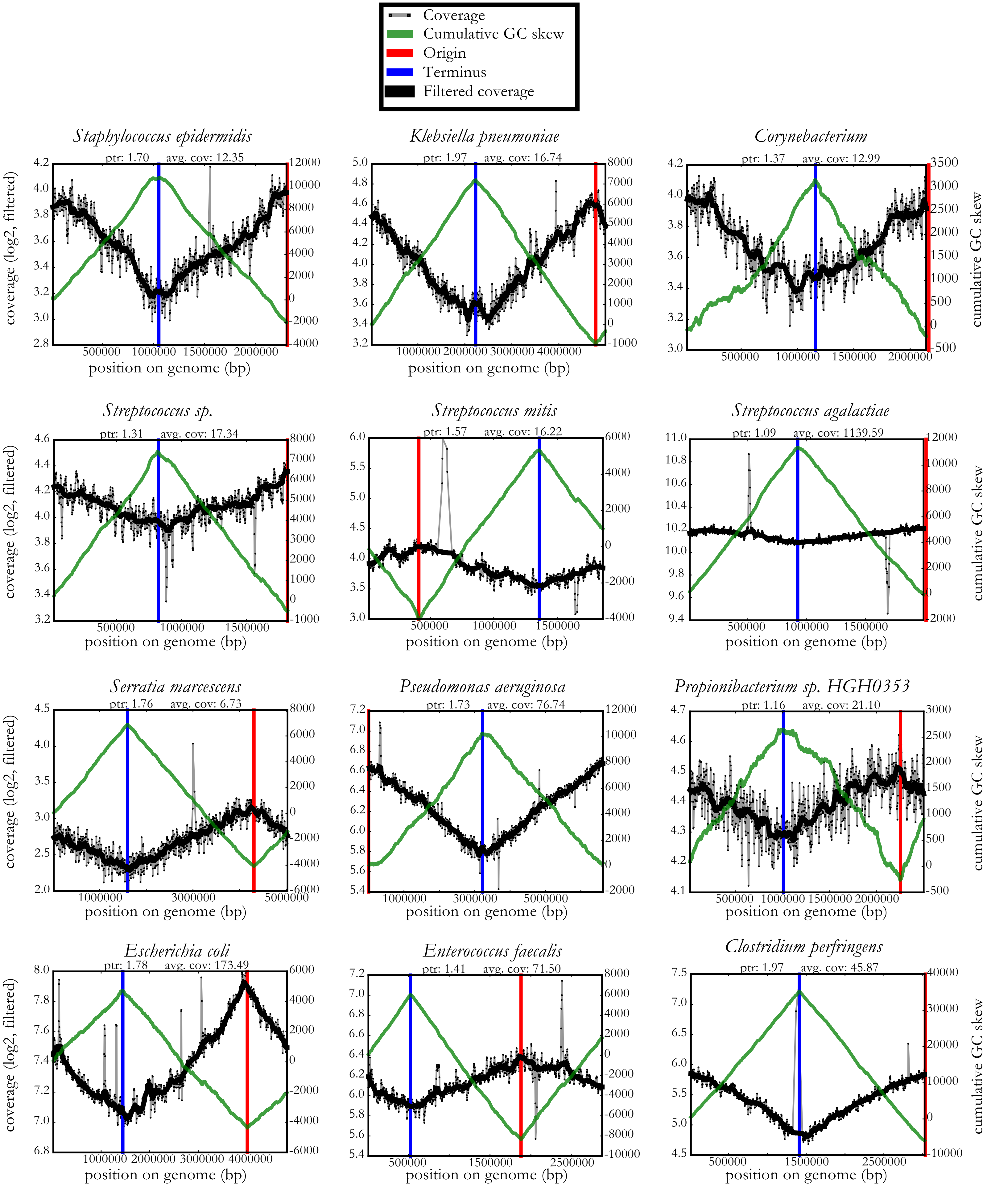


**Figure S6** Coverage and cumulative GC skew plots of ordered and oriented draft bacterial genomes recovered from Infant 2 samples. Both cumulative GC-skew and coverage support the same origin and terminus of replication, providing substantial evidence that ordering and orientation is correct.
