# Supplementary material for: Identical bacterial populations colonize premature infant gut, skin, and oral microbiomes and exhibit different in situ growth rates
Source: Genome Res. 2017 Apr;27(4):601–12. doi: 10.1101/gr.213256.116 (PMC5378178; doi:10.1101/gr.213256.116)
Supplement: Supplemental Material [file supp_gr.213256.116_Supplemental_Pileup.py.html]

Supplemental\_Pileup 

# Identical bacterial populations colonize premature infant gut, skin, and oral microbiomes and exhibit different in situ growth rates
